# Down-Regulation of Yes Associated Protein 1 Expression Reduces Cell Proliferation and Clonogenicity of Pancreatic Cancer Cells

**DOI:** 10.1371/journal.pone.0032783

**Published:** 2012-03-01

**Authors:** Caroline H. Diep, Kelly M. Zucker, Galen Hostetter, Aprill Watanabe, Chengcheng Hu, Ruben M. Munoz, Daniel D. Von Hoff, Haiyong Han

**Affiliations:** 1 Clinical Translational Division, Translational Genomics Research Institute, Scottsdale, Arizona, United States of America; 2 Integrated Cancer Genomics Division, Translational Genomics Research Institute, Scottsdale, Arizona, United States of America; 3 Epidemiology and Biostatistics Division, College of Public Health, University of Arizona, Tucson, Arizona, United States of America; Sanford Burnham Medical Research Institute, United States of America

## Abstract

**Background:**

The Hippo pathway regulates organ size by inhibiting cell proliferation and promoting cell apoptosis upon its activation. The Yes Associated Protein 1 (YAP1) is a nuclear effector of the Hippo pathway that promotes cell growth as a transcription co-activator. In human cancer, the YAP1 gene was reported as amplified and over-expressed in several tumor types.

**Methods:**

Immunohistochemical staining of YAP1 protein was used to assess the expression of YAP1 in pancreatic tumor tissues. siRNA oligonucleotides were used to knockdown the expression of YAP1 and their effects on pancreatic cancer cells were investigated using cell proliferation, apoptosis, and anchorage-independent growth assays. The Wilcoxon signed-rank, Pearson correlation coefficient, Kendall's Tau, Spearman's Rho, and an independent two-sample *t* (two-tailed) test were used to determine the statistical significance of the data.

**Results:**

Immunohistochemistry studies in pancreatic tumor tissues revealed YAP1 staining intensities were moderate to strong in the nucleus and cytoplasm of the tumor cells, whereas the adjacent normal epithelial showed negative to weak staining. In cultured cells, YAP1 expression and localization was modulated by cell density. YAP1 total protein expression increased in the nuclear fractions in BxPC-3 and PANC-1, while it declined in HPDE6 as cell density increased. Additionally, treatment of pancreatic cancer cell lines, BxPC-3 and PANC-1, with YAP1-targeting siRNA oligonucleotides significantly reduced their proliferation *in vitro*. Furthermore, treatment with YAP1 siRNA oligonucleotides diminished the anchorage-independent growth on soft agar of pancreatic cancer cells, suggesting a role of YAP1 in pancreatic cancer tumorigenesis.

**Conclusions:**

YAP1 is overexpressed in pancreatic cancer tissues and potentially plays an important role in the clonogenicity and growth of pancreatic cancer cells.

## Introduction

As one of the most lethal forms of human disease, pancreatic cancer has a one-year and five-year survival rate of 26% and 6%, respectively [1]. There exists a critical need for novel treatment strategies to provide a purposeful effect on the survival of patients with pancreatic cancer.

The Hippo pathway regulates organ size by inhibiting cell proliferation and promotes cell apoptosis [2], [3]. Components of Hippo pathway were identified in genetic mosaic screens in *Drosophila*, which consist of the Hippo (Hpo) kinase, the Salvador (Sav) adaptor protein, the Wts protein kinase, the Mats adaptor protein, and the Yorkie (Yki) nuclear transcriptional co-activator. Upon pathway activation, Hpo and Sav phosphorylates and activates Wts in conjunction with Mats. Wts phosphorylates Yki to inactivate and retain it in the cytoplasm. In mammals, the components of the Hippo pathway are highly conserved homologs, which include MST1/2 (Hpo), WW45 (Sav), LATS1/2 (Wts), MOB1 (Mats), and YAP1 (Yki) [4]–[8].

Similar to the *Drosophila* model, the mammalian Hippo core components form protein kinase complexes acting in a cascade to phosphorylate YAP1 (also known as YAP or YAP65) and relocate it to the cytoplasm [6], [7], [9], [10]. As the major downstream target of the Hippo pathway, YAP1 is a paradox. As an oncogene, the amplification of the YAP1 gene locus at 11q22 is found in several cancer types, including hepatocellular carcinoma, breast cancer, oral squamous cell carcinomas, medulloblastomas, and esophageal squamous cell carcinomas [11]–[15]. In addition, overexpression of YAP1 protein and its nuclear localization have been noted in colon, liver, lung, ovarian, and prostate cancers [6], [12], [16]. Overholtzer and colleagues reported that the overexpression of YAP1 in an immortalized epithelial cell line MCF10A resulted in its oncogenic transformation [11]. In contrast, YAP1 was also found to stabilize and enhance p73-dependent apoptotic cell death during cisplatin-induced DNA damage [17]. AKT, a key player in multiple cellular survival pathways, has been shown to phosphorylate YAP1 in order to suppress pro-apoptotic gene expression [18]. In a subset of breast cancers, the YAP1 protein expression was significantly decreased due to loss of heterozygosity, and shRNA knockdown of YAP1 increased migration, invasiveness, and enhanced tumor growth [19]. Overall, these findings suggest that YAP1's expression and role in cancer might be cell type and/or cellular context dependent.

In this study, we sought to elucidate the role of YAP1 in pancreatic cancer. We examined the YAP1 protein expression and localization in pancreatic tumor tissues taken from patients with pancreatic cancer and investigated the phenotypic effects of YAP1 down-regulation in cultured pancreatic cell lines. We have determined that YAP1 is overexpressed in pancreatic tumor tissues, and the down-regulation of YAP1 abated proliferation and clonogenicity of cultured pancreatic cancer cells.

## Materials and Methods

### Cell Culture

The pancreatic cancer cell lines AsPC-1, BxPC-3, Capan-1, CFPAC-1, HPAF-II, Hs 766T, MIA PaCa-2, and PANC-1 were obtained from the American Type Culture Collection (ATCC) (Manassas, VA). The cell lines were maintained in RPMI 1640 (Invitrogen, Carlsbad, CA) supplemented with 10% fetal bovine serum (FBS) (Gemini Bio-Products, Woodland, CA), penicillin (100 U/ml), and streptomycin (100 mg/ml) (Invitrogen). The immortalized human pancreatic ductal epithelial cell line, HPDE6, was provided by Dr. M. S. Tsao (University of Toronto, Canada) and cultured in keratinocyte-serum free media supplemented with bovine pituitary extract (30 µg/ml) and epidermal growth factor (0.2 ng/ml) (Invitrogen) [20], [21]. The immortalized human pancreatic ductal epithelial cell line, hTERT-HPNE, was obtained from ATCC and cultured in DMEM media supplemented with 20% FBS [22]. All cells were routinely cultivated in a humidified incubator at 37°C and 5% CO_2_. Cell line identities were verified as previously described [23].

### Tissue Microarray and Immunohistochemistry (IHC)

A tissue microarray (TMA) was constructed from paraffin-embedded blocks of 66 unique cases of pancreatic ductal adenocarcinomas, 5 cases of chronic pancreatitis, and 6 normal pancreas samples as previously described [24]. For each cancer case, two tumor cores and one adjacent normal core were punched for the TMA. The TMA blocks were sectioned at 5 µm thickness, transferred by water flotation, and dried overnight at room temperature. The slides were dewaxed, rehydrated and antigen retrieved on-line on the BondMax™ autostainer (Leica Microsystems, Inc Bannockburn, IL). All slides were subjected to heat induced epitope retrieval using an EDTA based retrieval solution (Leica Microsystems) for 20 minutes. Endogenous peroxidase and biotin were blocked. The TMA sections were incubated for 30 minutes at 1∶125 with YAP1 (H-125) antibody from Santa Cruz Biotechnology, Inc (Santa Cruz, CA). The sections were visualized using the Bond™ Polymer Refine Detection kit (Leica Microsystems) using diaminobenzidine chromogen as substrate and scored as previously described [24]. The Wilcoxon signed-rank test was used to compare level of YAP1 expression between the tumor and its adjacent normal samples. The Wilcoxon rank-sum test was used to compare the YAP1 expression between tumor and pancreatitis as well as normal pancreas samples from a separate group of subjects. The Pearson's correlation coefficient was used to estimate and test the association between nuclear YAP1 expression and histopathological parameters (tumor grade, stage, and disease in regional lymph nodes), and sensitivity analysis was carried out using Kendall's tau and Spearman's rho.

### YAP1 Immunoblotting Analysis

To achieve the necessary cell density after 48 hours of growth, BxPC-3, PANC-1, and HPDE6 cells were seeded in the following manner in T175 cm^2^ flasks in complete growth media: two million cells for 20–30%, four million cells for 50–70%, and seven million cells for 100%. The cells were washed with DPBS, trypsinized, and then counted on the Cellometer Auto T4 (Nexcelcom Biosciences, Lawrence, MA). Four million cells were used for sub-cellular fractionation using the BioVision Nuclear/Cytosolic Fractionation Kit (Mountain View, CA) by following the manufacturer's protocol. To generate whole cell lysates one million cells were lysed in RIPA buffer (Cell Signaling Technology, Danvers, MA) with 1X protease and phosphatase inhibitor cocktail (Roche) and incubated in ice for 30 minutes. The extracts were centrifuged at 14,000× g for 10 minutes at 4°C. Protein concentration was determined using the bicinchoninic acid (BCA) protein assay (Pierce Biotechnology, Rockford, IL). A total of 20 µg of protein per lane was separated by 4–12% NuPAGE Bis-Tris gel electrophoresis (Invitrogen). The blots were incubated with either a Phospho-YAP1 (Ser127) antibody (Cell Signaling) at 1∶2000 dilution or a total YAP1 (H-125) antibody (Santa Cruz Biotechnologies, Santa Cruz, CA) at 1∶2000 dilution overnight at 4°C and then exposed to anti-rabbit IgG HRP-linked antibody (Cell Signaling) at 1∶3000 dilution at room temperature for 1 hour. PARP (Cell Signaling) at 1∶2000 dilution served as the loading control for the nuclear fraction, while α-tubulin (Cell Signaling) at 1∶2000 dilution served as a control for the whole cell lysates and cytosolic fraction.

### siRNA treatment and Cell Proliferation Assays

YAP1 expression silencing was achieved with two YAP1 siRNA duplex oligonucleotides targeting two different regions of YAP1 transcript (Qiagen, Valencia, CA) at a final concentration of 20 nM using the siLentfect transfection reagent (Bio-Rad) according to the manufacturer's protocol. The siRNAs were reverse-transfected by incubating the siRNAs in 10 µl serum-free RPMI 1640 cell culture media (Invitrogen) containing 50 µl of the siLentfect lipid reagent (Bio-Rad) mix per well and arrayed into 96-well plates. The siRNAs and transfection reagent were allowed to incubate at room temperature for 30 minutes. Afterwards, the cells were plated to the siRNA-transfection reagent mix at 4800 cells/well and serum-supplemented at a final concentration of 5% for BxPC-3 and 2% for PANC-1. For the HPDE6 cell line, 6000 cells/well were plated to siRNA-transfection reagent mix in the keratinocyte medium. The plates were stored in a humidified incubator at 37°C and 5% CO_2_. After 24 hours, the transfection media was removed from each well and replaced with fresh serum-supplemented growth media. Cell viability was determined by adding 100 µl of the CellTiter-Glo Luminescent Assay (Promega, Madison, WI) to each well. The plates were incubated at 37°C for one hour, and the luminescence was recorded with the Synergy HT microplate reader (BioTek, Winooski, VT).

### Apoptosis and cell cycle analysis

The transfection of YAP1 siRNAs into the pancreatic cells lines was as described in the cell proliferation studies above. Ninety-six hours after initial transfection, caspase-3 and −7 activities were determined by adding 100 µl of the Caspase-Glo 3/7 Assay (Promega) to each well. The plates were incubated at 37°C for one hour, and the luminescence was recorded with the Synergy HT microplate reader (BioTek). The caspase activity in cells treated with siRNA was normalized to that of the Cells Only control.

Cell cycle analysis of YAP1 siRNA treated pancreatic cell lines were as previously described [23].

### Anchorage-independent assays

In 6-well plates, 250,000 cells (BxPC-3 and PANC-1) were reverse transfected with 20 nM of the YAP1 siRNAs (Qiagen) for 48 hours (and 72 hours for protein extraction and immunoblot analysis). Cells were washed with DPBS, trypsinized, and counted by trypan blue exclusion. Six thousand viable BxPC-3 cells and 3,000 viable PANC-1 cells were mixed with 1 ml of growth media and 0.3% agar and then layered onto 0.5% agar beds in 35 mm grid dishes. Cells were allowed to grow for 21 days and the entire area of the dish was counted. Colonies larger than 50 cells were counted as positive for transformation. Assays were conducted in triplicate.

Cells remaining from the transfection were kept for RNA and protein extraction. For RNA extraction, the RNeasy Mini Kit (Qiagen) was utilized following the manufacturer's recommended protocol. One microgram of total RNA was used in a 20 µl cDNA synthesis reaction (Quantas Biosciences, Gaithersburg, MD). Reactions were carried out in 1 µl of the cDNA reaction mix, 10 µl SYBR Green/Taq Polymerase master mix (Roche, Indianapolis, IN), 4 µl of primers (YAP1, 5′-GCCATTAAAGGCAGCTGTTC-3′ and 5′-AGCACTGTGCCAGGTATCAC-3′; GAPDH, 5′-ATTGCCCTCAACGACCACTT-3′ and 5′-GGTCCACCACCCTGTTGC-3′, at a final concentration 400 nM), and 5 µl water to a final volume of 20 µl. The Bio-Rad MyIQ single color real-time PCR detection system (Hercules, CA) was used to perform the RT-PCR. Two-step amplification (95°C for 30 sec and 58°C for 30 sec) was repeated for 40 cycles. Following the PCR reaction, a melting curve analysis was performed (60°C for 5 sec) for 35 cycles. The data was analyzed using the comparative C_T_ method [25]. Analysis of whole cell lysate protein level expression of YAP1 was as previously described, with the exception that the siRNA-treatment was for 72 hrs.

## Results

### YAP1 is overexpressed in pancreatic ductal adenocarcinomas (PDA)

To evaluate the expression level of YAP1 protein in PDA tissues, we performed immunostaining using a tissue microarray (TMA). Among the 66 PDA cases contained in the TMA, 64 were evaluable for tumor staining, of which 33 had matching normal epithelium. The YAP1 antibody showed both nuclear and cytoplasmic staining, which is consistent with the known cellular function of YAP1 protein – YAP1 is localized in cytoplasm when it is inactivated and it is translocated into nucleus when activated. We assessed the staining intensity for both nuclear and cytoplasmic YAP1 and summarized the scores in Table 1. Approximately 77% (49/64) of the PDA cases had positive (scored 1+ or higher) nuclear YAP1 staining in tumor cells, 63% (31/49) of which had moderate (2+) or strong (3+) staining. In contrast, only 48% (16/33) of the cases had positive staining in the adjacent normal epithelium, with only 18% (6/33) had moderate or strong staining. On the other hand, the staining intensities in the cytoplasm are comparable between the tumor and adjacent normal tissues, where 56% (36/64) of the cases had 2+ or higher staining in the tumor and 43% (14/33) had 2+ or higher staining in adjacent normal epithelium. Analysis of the tumor cases with matching adjacent normal tissues using Wilcoxon signed-rank test also showed significantly higher expression in the tumor cells compared to adjacent normal ductal cells with a p-value of 0.0002. But more importantly, the difference in YAP1 protein level between tumor and adjacent normal epithelium is much more significant in the nucleus (p-value = 0.0007) than in the cytoplasm (p-value = 0.027), indicating that YAP1 is not only overexpressed in PDA but it is also highly activated.

**Table 1:** Summary of YAP1 immunostaining in pancreatic tumors and adjacent normal samples.

YAP1 Nuclear Expression				
Staining Intensity	Tumor	Percent	Adjacent Normal	Percent
Negative = 0	15	23.4%	17	51.5%
Weak = 1	18	28.1%	10	30.3%
Moderate = 2	19	29.7%	4	12.1%
Strong = 3	12	18.8%	2	6.1%
n =	64		33	

YAP1 Cytoplasm Expression				
Staining Intensity	Tumor	Percent	Adjacent Normal	Percent
Negative = 0	4	6.3%	2	6.1%
Weak = 1	24	37.5%	17	51.5%
Moderate = 2	26	40.6%	12	36.4%
Strong = 3	10	15.6%	2	6.1%
n =	64		33	

**Figure 1:**
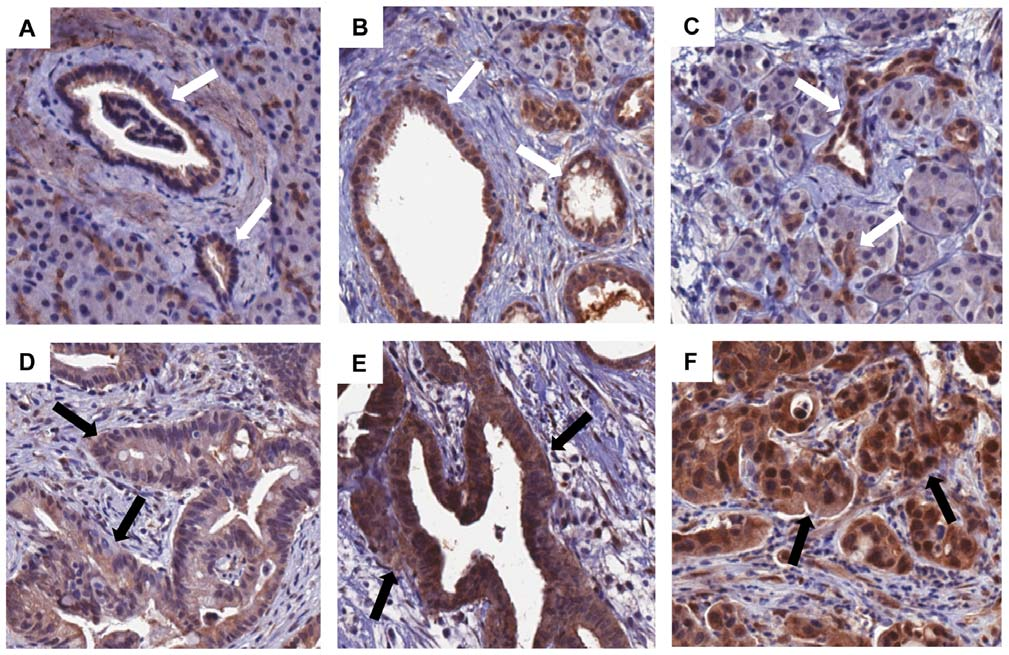
Immunohistochemical staining of YAP1 in pancreatic tumor samples. A) Negative to weak cytoplasmic staining in normal ductal epithelium (white arrows). B) Moderate staining (mostly cytoplasmic) in normal ducts adjacent to tumor (white arrows). C) Moderate to strong staining in a normal centroacinar and small ductal cells (white arrows); D) Weak staining in a ductal adenocarcinoma (black arrows); E) Strong staining in a well differentiated ductal adenocarcinom (black arrows); and F) strong staining (mostly nuclear) in a poorly differentiated ductal adenocarcinoma (black arrows).

Figure 1 depicts examples of differential YAP1 staining in PDA, adjacent normal tissues, and normal pancreas. In some normal pancreas and pancreatitis tissues we observed moderate to strong YAP1 staining in the acinar cells and small ducts (Figure 1C). Nonetheless, the overall expression of YAP1 in the tumor is significantly higher than in the normal pancreas and pancreatitis cases (p-value = 0.011).

Next, we correlated the activated nuclear YAP1 expression level with histopathological parameters in patients with pancreatic cancer. As shown in Table 2, there is no association between nuclear YAP1 expression and tumor grade or residual disease in regional lymph nodes, but there exists a marginally significant correlation (r = 0.33 and p-value = 0.068 in the Pearson correlation coefficient analysis) between nuclear YAP1 level and tumor stage.

**Table 2:** Nuclear YAP1 expression level and its correlation with histopathological parameters in pancreatic cancer.

# Patients Parameters		Nuclear YAP1 expression				Pearson Correlation Coefficient	p-value	Kendall's Tau	p-value	Spearman's Rho	p-value
		0	1+	2+	3+						
Grade	1	2	2	1	0	−0.157	0.398	−0.121	0.451	−0.142	0.445
	2	4	7	5	4						
	3	4	1	1	0						
Stage	I	1	0	0	0	0.332	0.068	0.291	0.079	0.317	0.082
	II	3	3	1	0						
	III	6	7	6	4						
	IV	0	0	0	0						
pN	0	4	6	2	2	0.016	0.93	0.014	0.934	0.015	0.936
	1	6	4	5	2						

Abbreviation: pN, pathological nodal status; 0 = negative, 1 = metastasis present.

**Figure 2:**
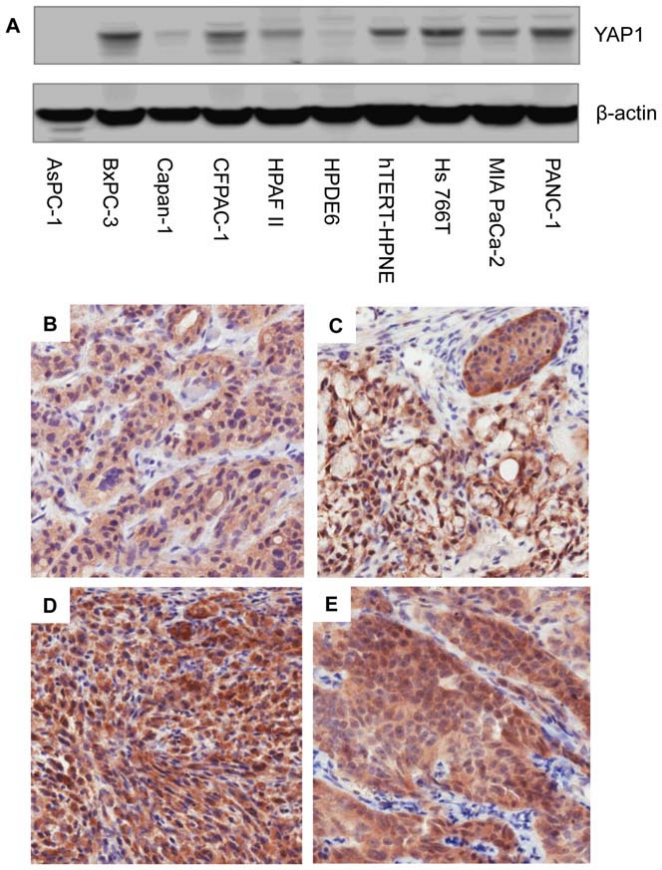
YAP1 expression in pancreatic cell lines and xenograft tumors. A) Western blotting analysis of YAP1 protein expression in a panel of eight pancreatic cancer and two immortalized (non-transformed) pancreatic cell lines. B–E) Immunohistochemical staining for YAP1 in pancreatic cancer xenografts: B) AsPC-1; C) BxPC-3; D) MIA PaCa-2; and E) PANC-1.

We also examine the expression level of YAP1 in a panel of eight human pancreatic cancer and two immortalized normal human pancreatic epithelial cell lines using Western blotting (Figure 2A). Six pancreatic cancer cell lines (BxPC-3, CFPAC-1, HPAF-II, Hs 766T, MIA PaCa-2, and PANC-1) exhibited high to moderate protein levels of YAP1. Two pancreatic cancer cell lines (AsPC-1 and Capan-1) had undetectable to low YAP1 protein levels, respectively. The two immortalized pancreatic cell lines (HPDE6 and hTERT-HPNE) had low to moderate YAP1 protein expression. This expression profile of YAP1 in pancreatic cell lines is consistent with what we observed in the pancreatic tumor tissue samples (Table 1).

Further investigation of YAP1 expression in xenograft tumors formed by the pancreatic cancer cell lines also showed similar results. Figure 2 depicts the differential staining and localization of YAP1 in xenograft tumors of four pancreatic cancer cell lines using immunohistochemistry. The AsPC-1 tumor had very low staining of YAP1 and was limited to the cytoplasm (Figure 2B), which is in accordance to the Western blotting results (Figure 2A). BxPC-3 and MIA PaCa-2, which had moderate to high protein expression of YAP1 by Western blotting (Figure 2A), showed moderate to strong immunostaining in the cytoplasm and moderate (sometimes strong) staining in the nuclei (2C and 2D, respectively). PANC-1 had similar YAP1 protein expression with intense staining of the cytoplasm with low to moderate staining of the nuclei (Figure 2E).

### The expression and localization of YAP1 is regulated by cell density

It has been reported that increased cell density can result in the activation of Hippo pathway and subsequently the sequestering of YAP1 in the cytoplasm [6]. We hypothesized that in pancreatic cancer cells such regulation of YAP1 localization by cell density is diminished. To explore this hypothesis, we assessed the expression level and localization of YAP1 at varying cell densities by subcellular protein fractionation and immunoblotting.

**Figure 3:**
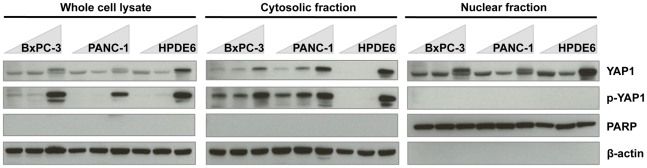
YAP1 protein expression and localization in pancreatic cancer cell lines. As cell density increases, the pancreatic cancer cell lines, BxPC-3 and PANC-1, response to the deactivation of YAP1 as a transcription cofactor by cytosolic sequestration is less substantial than HPDE6. Cells were extracted at increasing cell densities after 48 hours of initial seeding. PARP serves as the loading control for the nuclear fraction. α-tubulin serves as the loading control for the whole cell lysates and cytosolic fraction.

As shown in Figure 3, at lower cell densities (20–70%), the YAP1 protein was present in both the cytoplasm and nucleus in the two pancreatic cancer cell lines, BxPC-3, and PANC-1, whereas in the immortalized normal pancreatic ductal epithelial cell line, HPDE6, YAP1 was not detectable in the cytosolic fraction. When cells became 100% confluent, the relative nuclear YAP1 protein level (ratio of nuclear YAP1 vs. total YAP1) increased in BxPC-3 and PANC-1, but not in HPDE6 (Figure S1). Although the cytosolic YAP1 protein level increases as the cells become confluent in all three cell lines, the increase in HPDE6 is the most significant. This indicates that relatively more YAP1 remain activated (unphosphorylated) in the cancer cell lines than in the immortalized normal HPDE6 cell line when cells become confluent. The level of phospho-YAP1 (p-YAP1) protein, which is only detected in the whole cell lysate and the cytosolic fraction in the cell lines, also confirms the differences in YAP1 cellular localization between the cancer cell lines and HPDE6. p-YAP1 was not detected in the cytosolic fraction of low confluent HPDE6 cells and there was a dramatic increase when the cells became 100% confluent. In the cancer cells, on the other hand, p-YAP1 is present at low cell densities and increases as the cells become more confluent. These results indicate that YAP1 expression and localization is not as tightly regulated by cell density in cancer cells as in the normal cells, suggesting that YAP1 function might be dysregulated in pancreatic cancer cells.

### siRNA knockdown of YAP1 reduces cell proliferation, induces apoptosis, and abates anchorage-independent growth in pancreatic cancer cell lines

To further analyze the role of YAP1 in tumorigenesis, we examined the effect of YAP1 silencing by siRNA oligonucleotides on pancreatic cell proliferation, apoptosis, and anchorage-independent growth. BxPC-3 and PANC-1 cells were reverse-transfected with two siRNA oligonucleotides targeting different regions of the YAP1 mRNA sequence. The AllStars Negative Control siRNA (Neg siRNA) and AllStars Death Control siRNA (Qiagen) were used as controls to compare siRNA transfection effects on cell proliferation and transfection efficiency.

**Figure 4:**
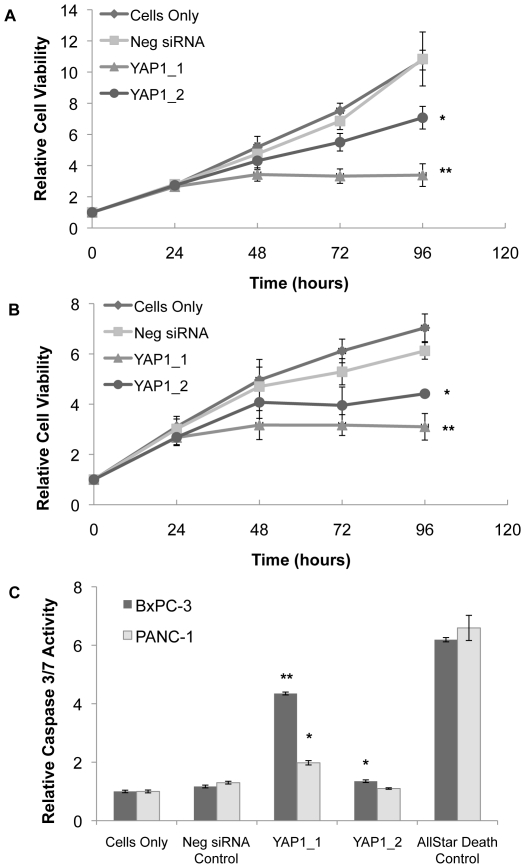
Effect of YAP1 targeted siRNAs on the proliferation and apoptosis of pancreatic cancer cell lines. The cell growth curves of A) BxPC-3 and B) PANC-1 transfected with YAP1 siRNA oligonucleotides. The independent two-sample *t* test (two-tailed) was utilized to calculate the statistical significance at the 96-hour time point compared to Neg siRNA. * denotes p<0.05, and ** denotes p<0.01. C) The Caspase-Glo 3/7 Assay (Promega) was used to determine the level of apoptosis in pancreatic cancer cells transfected with YAP1 siRNA oligonucleotides after 72 hours. ** denotes an independent two-sample *t* test (two-tailed) p-value of <0.01 when compared to the negative siRNA control (Neg siRNA).

Over a time course of 96 hours, cells treated with YAP1 siRNA had significant reduction in growth rates compared to the Cells Only and negative siRNA (Neg siRNA) control. In Figure 4, the Neg siRNA had a minimal effect on the proliferation of BxPC-3 and PANC-1 compared to the untreated Cells Only control. However, both YAP1 siRNA sequences had significantly suppressed proliferation in both BxPC-3 and PANC-1. Ninety-six hours after siRNA treatment, siRNA sequence YAP1_1 inhibited the growth of BxPC-3 and PANC-1 cells by 69% and 53%, respectively (p<0.01) and the siRNA sequence YAP1_2 inhibited the growth by 34% and 33%, respectively (p<0.05). YAP1 siRNA sequences did not significantly reduce proliferation in HPDE6 (Figure S2A).

To evaluate the effect of YAP1 inhibition on apoptosis, we measured the activation of caspase-3 and caspase-7 in pancreatic cancer cells treated with siRNA. As shown in Figure 4C, 72 hours after transfection, both YAP1 siRNA sequences induced caspase 3/7 activity by more than 2-fold (p<0.05) when compared to the negative siRNA control in BxPC-3, while one sequence (YAP1_1) significantly induced caspase activity (p<0.05) in PANC-1. However, neither YAP1 siRNA sequence significantly induced caspase 3/7 activity in the HPDE6 cells (Figure S2B). This finding suggests that knockdown of YAP1 expression induces caspase-dependent apoptosis in the pancreatic cancer cells.

Next, the effects of YAP1 inhibition on cell cycle distribution were examined (Table S1). Both YAP1 siRNA sequences caused a considerable increase in the G0/G1 cell population in both BxPC-3 and PANC-1, comparing to the negative siRNA control. In HPDE6, the two siRNA sequences did not induce consistent effects on cell cycle distribution when compared to the Neg siRNA control (Table S1).

**Figure 5:**
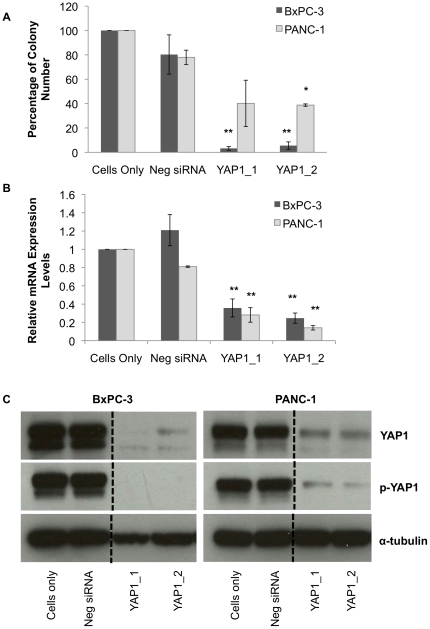
Inhibition of anchorage-independent growth of pancreatic cancer cells by YAP1 siRNA oligonucleotides. A) Percentage of colonies relative to the Cells Only control on soft agar after 21 days of growth from initial seeding. p-values were calculated by comparing the YAP1 siRNA (YAP1_1 and YAP1_2) to the Neg siRNA (independent two tailed *t* test, two-tailed). B) qPCR analysis of YAP1 mRNA expression after 48 hours of siRNA transfection. C) Western blotting analysis of YAP1 expression after siRNA-treatment for 72 hrs. The samples for each cell line were separated and analyzed on the same blot.* denotes p<0.05, and ** denotes p<0.01.

Since we observed a significant effect on cell proliferation, we wanted to consider if YAP1 knockdown by siRNA would abolish anchorage-independent growth of pancreatic cancer cells. BxPC-3 and PANC-1 cells were treated with the siRNA sequences for 48 hrs and harvested by trypsinization. Equal number of viable cells for each treatment was then seeded on soft agar. After 21 days, colonies were counted from each group and then normalized to the Cells Only control and represented as a percentage of colonies. Both YAP1_1 and YAP1_2 siRNAs suppressed clonogenicity in soft agar in BxPC-3 (95%, p<0.01) and in PANC-1 (60%, p<0.05) (Figure 5A). In contrast to the similar inhibitory activity in the cell proliferation assay (Figure 4A and 4B), the reduction in colony formation was much greater in BxPC-3 cells (∼95%) than that in PANC-1 cells (∼60%). This difference is consistent with the level of YAP1 mRNA and protein reduction by the siRNA oligonucleotides in the cell lines (Figure 5B and 5C). As shown in Figure 5C, the reduced protein expression by YAP1 siRNAs is more dramatic in BxPC-3 cells than in PANC-1 cells.

## Discussion

Originally discovered in Drosophila as a potent pathway for tumor suppression, the Hippo pathway negatively regulates cell growth through a kinase cascade and results in the inactivation of Yki (YAP1 in mammals) [6], [7], [9], [10]. The pathway and its components are highly conserved in mammals, and multiple studies have demonstrated the pathway's compelling roles of regulation of cell growth, proliferation, survival, and association in malignancies [26]–[33]. As the nuclear effector of transcription, YAP1 has become an attractive target of investigation in mammalian malignancies. In our study, we have observed that YAP1 is overexpressed in pancreatic tumors and in cancer cell lines. The down-regulation of YAP1 diminished cell viability, proliferation, and anchorage-independent growth in cultured cells.

The subcellular localization of YAP1 in the pancreatic tumors and in the cultured cell lines highlighted the dysregulation of the Hippo pathway. Upon pathway activation, YAP1 is inactivated via phosphorylation by LATS1/2 and consequently retained in the cytoplasm [6], [7]. In our immunostaining studies, YAP1 staining is generally more intense in the cytoplasm than in the nucleus, and the tumor has a significantly higher proportion of cells with positive YAP1 staining in the nucleus than the adjacent normal (77% vs. 48%). Although a significant percentage of adjacent normal cases stained positive for YAP1, the overall expression level is significantly higher in the tumor (Table 1). The staining of YAP1 in normal pancreas tissues is restricted to acinar cells and small ducts, which is consistent with a previous study [34] and likely represents the normal function of YAP1 in maintaining tissue homeostasis.

As a previous study [6] and ours have observed, YAP1 protein expression is modulated by increasing density. At low density, YAP1 protein expression is minimal, but as cell density increases YAP1 expression increases accordingly. In Figure 3, a slower migrating band was observed in the pancreatic cancer cell lines immunoblotted for total YAP1 when cell density reached 100% in the whole cell lysate and nuclear fractions. We postulate that the slower migrating band observed in the nuclear fraction is not due to the phosphorylation known to regulate YAP1 localization, but rather a post-translational modification unknown at this time [35]–[38]. For future studies, the cell density should be noted in relationship to YAP1 protein expression. In pancreatic cell lines, YAP1 protein expression and localization is regulated by cell density, but such regulation seemed to be diminished significantly in cancer cells compared to that in normal ductal epithelial cells (Figure 3). Interestingly, although we see high levels of YAP1 staining in both nucleus and cytoplasm in PDA tissues, the nuclear YAP1 protein level had more significant increase than the cytoplasmic YAP1 when compared to adjacent normal tissues (Table 1). This perhaps indicates that, in addition to the upregulation of YAP1 protein expression, components of the Hippo pathway that regulate YAP1 localization (phosphorylation) might also be dysregulated in pancreatic cancer. In fact, the LATS1/2 kinases upstream of YAP1 have been shown to be down-regulated in several cancer types including soft tissue sarcoma [39], astrocytoma [40], and breast cancer [41], [42]. Most recently, Zhou et al. reported the loss of active MST1, another kinase upstream of YAP1, in human hepatocellular carcinomas (HCC) and are tumor suppressive in transgenic mouse models [43]–[45]. Investigation of these upstream regulators of YAP1 in pancreatic cancer will shed further light on the role of Hippo pathway in the tumorigenesis and progression of pancreatic cancer.

Although the oncogenic activity of YAP1 has been clearly demonstrated [11], [12], data from some studies also indicate that YAP1 might be tumor suppressive in certain cell types. For example, it has been shown that activated nuclear YAP1 forms a complex with the p73 tumor suppressor protein and prevents the latter from being degraded, which, in turn, results in increased apoptosis. In our work, we demonstrate that suppression of YAP1 expression by siRNA reduced pancreatic cell growth and clonogenicity, which is consistent with the notion that YAP1 is an oncogene. Our findings are also in agreement with the results obtained from HCC models using YAP1 targeted shRNA, siRNA, and miRNA constructs [12], [28], [46]. It is possible that in different tissues and/or under different conditions, YAP1 interacts with different partners to exert different functions [27], [28]. In conclusion, our study shows that YAP1 is overexpressed in pancreatic cancer and offers a mechanistic insight into the phenotypic effect of the down-regulation of YAP1. YAP1 and its downstream effectors represent potential new therapeutic targets in pancreatic cancer.

## Supporting Information

Figure S1 **Quantification of YAP1 protein expression at different cell densities.** The total YAP1 protein in the nuclear fraction and the whole cell lysate in BxPC-3, PANC-1, and HPDE6 cells with increasing cell densities shown in Figure 3 were quantified by densitometry and normalized to the loading controls (nuclear = PARP; whole cell lysate = alpha-tubulin). The ratios of the normalized values of total YAP protein in the nuclear fraction to the whole cell lysate were calculated and graphed.
(TIF)

Figure S2 **Effect of YAP1 targeted siRNAs on the proliferation and caspase activation in HPDE6 cells.** A) The relative proliferation rates of HPDE6 cells after 72 and 96 hours of YAP1 siRNA treatment. B) Caspase 3/7 activity levels in HPDE6 cells treated with YAP1 siRNA oligonucleotides for 72 hours.
(TIF)

Table S1 **Effects of YAP1 siRNA treatment on cell cycle in pancreatic cell lines.** The cell cycle distribution was measured using FACS analysis 72 hours after the transfection of siRNA oligonucleotides in BxPC-3, PANC-1, and HPDE6 cell lines.
(DOCX)
