# Supplementary figures and images for: Down-Regulation of Yes Associated Protein 1 Expression Reduces Cell Proliferation and Clonogenicity of Pancreatic Cancer Cells
Source: PLoS One. 2012 Mar 1;7(3):e32783. doi: 10.1371/journal.pone.0032783 (PMC3291657; doi:10.1371/journal.pone.0032783)

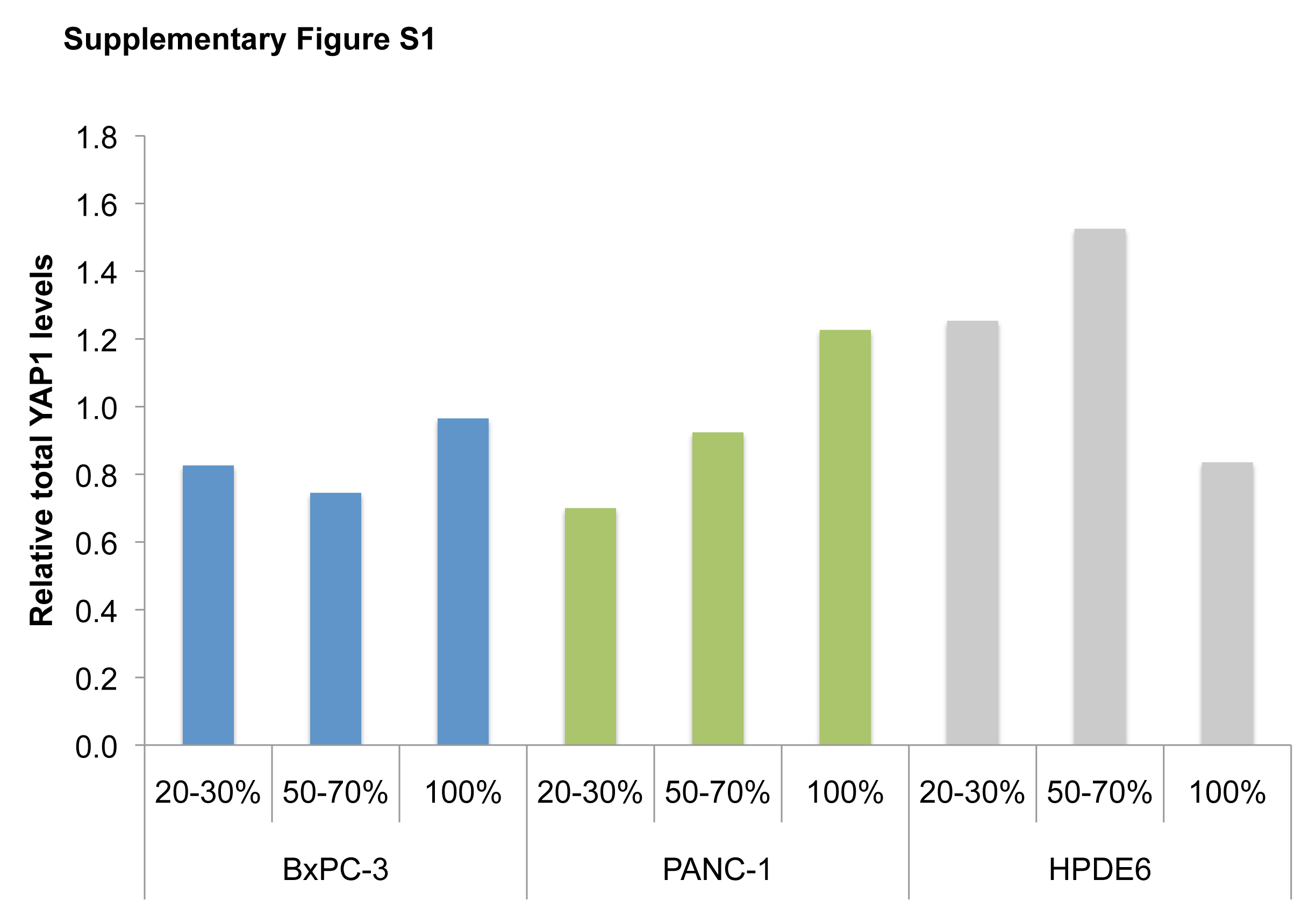

Supplement: Figure S1 — Quantification of YAP1 protein expression at different cell densities. The total YAP1 protein in the nuclear fraction and the whole cell lysate in BxPC-3, PANC-1, and HPDE6 cells with increasing cell densities shown in Figure 3 were quantified by densitometry and normalized to the loading controls (nuclear = PARP; whole cell lysate = alpha-tubulin). The ratios of the normalized values of total YAP protein in the nuclear fraction to the whole cell lysate were calculated and graphed. (TIF) [file pone.0032783.s001.tif]

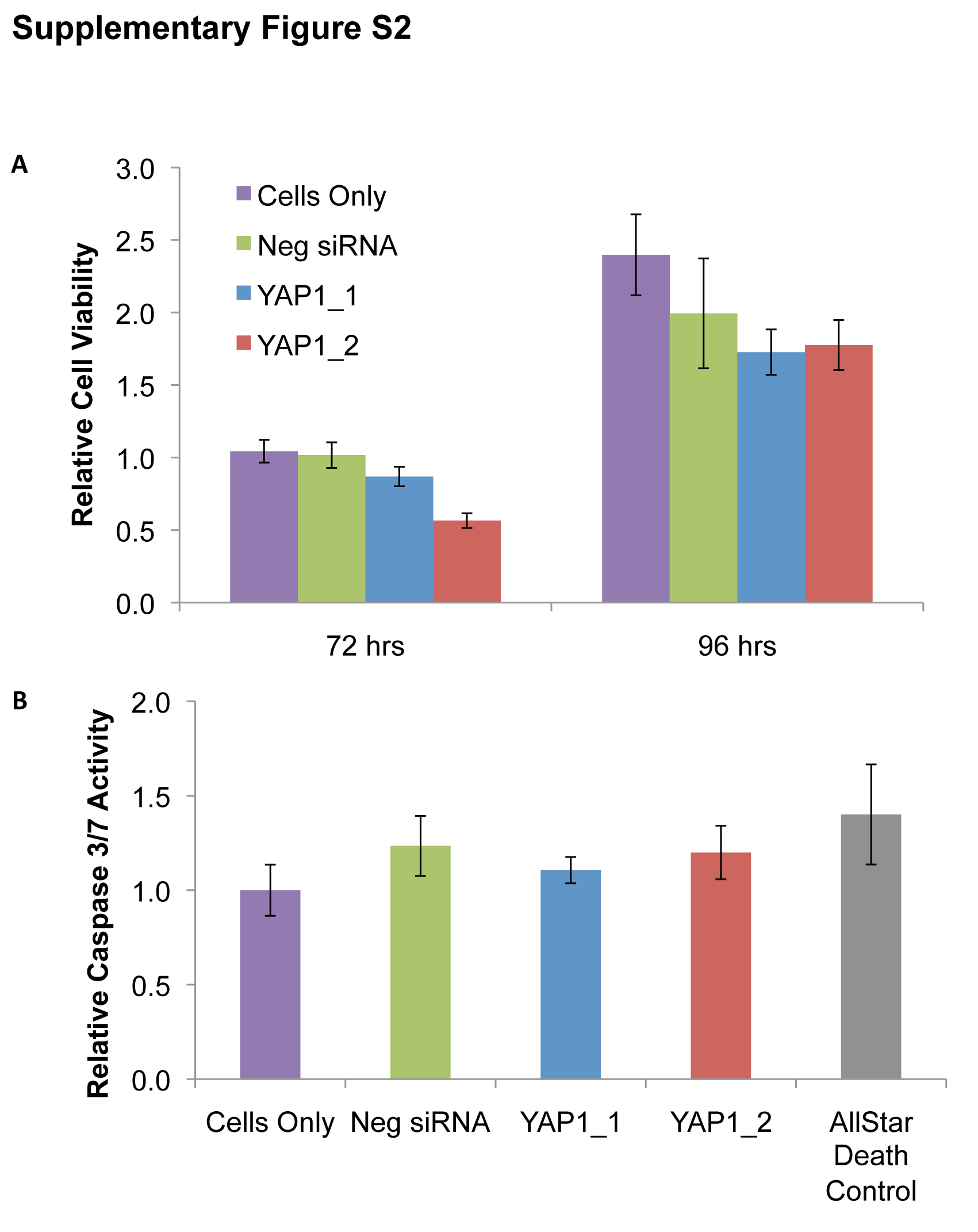

Supplement: Figure S2 — Effect of YAP1 targeted siRNAs on the proliferation and caspase activation in HPDE6 cells. A) The relative proliferation rates of HPDE6 cells after 72 and 96 hours of YAP1 siRNA treatment. B) Caspase 3/7 activity levels in HPDE6 cells treated with YAP1 siRNA oligonucleotides for 72 hours. (TIF) [file pone.0032783.s002.tif]
