# Supplementary material for: Down-Regulation of Yes Associated Protein 1 Expression Reduces Cell Proliferation and Clonogenicity of Pancreatic Cancer Cells
Source: PLoS One. 2012 Mar 1;7(3):e32783. doi: 10.1371/journal.pone.0032783 (PMC3291657; doi:10.1371/journal.pone.0032783)
Supplement: Table S1 — Effects of YAP1 siRNA treatment on cell cycle in pancreatic cell lines. The cell cycle distribution was measured using FACS analysis 72 hours after the transfection of siRNA oligonucleotides in BxPC-3, PANC-1, and HPDE6 cell lines. (DOCX) [file pone.0032783.s003.docx]

**Table S1. Effects of YAP1 siRNA treatment on cell cycle in pancreatic cell lines.**

|  |  | **G0/G1** | **S** | **G2/M** |
| --- | --- | --- | --- | --- |
|  | Cells only | 59.2 | 36.6 | 4.2 |
| **BxPC-3** | Neg siRNA | 64.5 | 29.9 | 5.6 |
|  | YAP1_1 | 69 | 26.5 | 4.6 |
|  | YAP1_2 | 71.4 | 25.4 | 3.2 |
|  |  |  |  |  |
|  |  | **G0/G1** | **S** | **G2/M** |
|  | Cells only | 62.5 | 31.4 | 6 |
| **PANC-1** | Neg siRNA | 53 | 36.4 | 10.6 |
|  | YAP1_1 | 64.1 | 21 | 15 |
|  | YAP1_2 | 77.2 | 20.2 | 2.6 |
|  |  |  |  |  |
|  |  | **G0/G1** | **S** | **G2/M** |
|  | Cells only | 52.7 | 40.7 | 6.6 |
| **HPDE6** | Neg siRNA | 71.6 | 23.9 | 4.5 |
|  | YAP1_1 | 77.9 | 17.6 | 4.6 |
|  | YAP1_2 | 64.4 | 28.8 | 6.8 |
